# An 81-million-word multi-genre corpus of Arabic books

**DOI:** 10.1016/j.dib.2025.111456

**Published:** 2025-03-09

**Authors:** Andreas Hallberg

**Affiliations:** University of Gothenburg, Department of Languages and Literatures, Box 200, 40530 Gothenburg, Sweden

**Keywords:** Arabic, Corpus linguistics, Genre

## Abstract

This article describes The Arabic E-Book Corpus, a freely available Arabic corpus consisting of 1,745 books (81,5 million words) published by the Hindawi Foundation between 2008 and 2024. The books are of various genres, including fiction and non-fiction, children's literature, plays, and poetry. Most of the texts are editions of works originally published in the 20th century, but the corpus also includes editions of older historical works. Books were retrieved in epub format and converted to plain text and html. Only books published under unrestricted licenses are included. Extensive metadata (were collected from colophons and the publisher's website title, author, genre, publication date, original publication date, original language, etc.). The corpus was originally collected in order to investigate variation in the use of vowel diacritics across genres, but it is also suitable for other linguistic inquiries, especially as relating to genre, and as a source of texts published under free licenses for training language models.

Specifications Table

**Table:** 

Subject	*Linguistics, Literature, Applied Machine Learning*
Specific subject area	Arabic corpus linguistic, especially as relating to genre.
Type of data	Text, Raw.
Data collection	Books were downloaded in epub format and converted to html and plain text. Only books published under free licenses were included. Metadata for each book were extracted from the books and their associated web pages*.*
Data source location	Swedish National Data Service
Data accessibility	Repository name: The Arabic E-Book Corpus [1] Data identification number: https://doi.org/10.5878/7rbhgy93 Direct URL to data: https://snd.se/sv/catalogue/dataset/2024-145/1#
Related research article	A. Hallberg, Principles of variation in the use of diacritics (*taškı̄l)* in Arabic books, Language Sciences. 93 (2022) 1–15. https://doi.org/10.1016/j.langsci.2022.101482/

## 1 Value of the Data

• This corpus is useful for linguistic investigations in Arabic, including in lexicon [2], orthography [3], diacritization [[4], [5], [6]], stylistics, especially as related to genre [7]; as well as for various fields in natural language processing, for example as free-licensed data for the development of large language models [8].
• In addition to collecting and formatting data for computational analysis, metadata was extracted and collected through a combination of automatic and manual methods, allowing for comparison of texts according to genre, date of publication, date of original publication, translated vs. untranslated works, etc.
• The corpus is available in two versions: plain text and html. The plain-text version is appropriate for most types of linguistic investigation. The html version includes markup of textual elements (foreign words, poetry, Quranic quotes, etc.), allowing for investigations that target these elements specifically.

## 2 Background

A previous version of this corpus was originally collected to investigate variation in the use of vowel diacritics across genres [5]. That study required a corpus of digital contemporary book-length texts of various genres. The version of the corpus investigated in that paper included texts with restricted licenses, meaning that the corpus could not be widely shared. In the version presented here, only works published under free licenses are included, allowing for the corpus to be freely accessible to researchers.

To the best of my knowledge, *The Arabic E-Book Corpus* is the only freely licensed Arabic corpus of contemporary book length publications. Other freely available Standard Arabic corpora are either collections of books primarily of traditional and historical Islamic literature [[9], [10], [11]] or of news articles [12,13]. The first is unsuitable for investigations in Modern Standard Arabic, and the latter is restricted to a single genre. See [14] for an overview.

For a similar dataset with the complete set of books by the same publisher (Hindawi), including works with non-free silences, see [15]. That dataset is twice the size of *The Arabic E-Book Corpus,* but with the rights to the additional books held by the original publisher, the author, or the author's estate. In addition, the current dataset is enriched with metadata lacking in [15].

## 3 Data Description

### 3.1 Contents and coverage

**Fig. 1:**
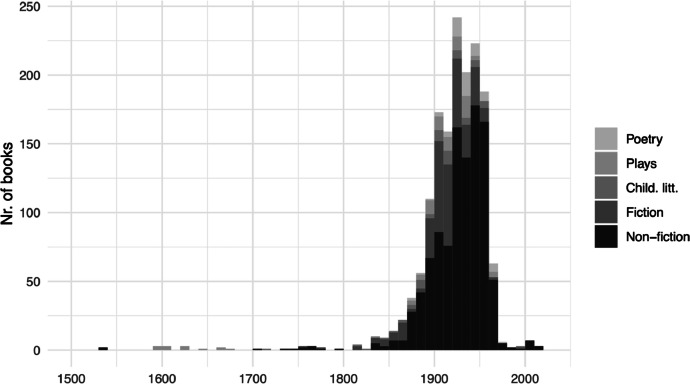
Original publication date. Thirteen books originally published between 724 and 1500 are excluded in the graph.

The corpus consists of 1, 745 books published between 2008 and 2024 by the Hindawi Foundation as free e-books, to a total of 81, 5 million words. Of these, 491 books are works translated to Arabic from other languages, primarily from English and French (see Table 1). As shown in Fig. 1, most of the books are editions of works originally published in the early and mid-20th century, but the corpus also includes historical material.^1^

**Table 1:** Translated works by original language.

English	372
French	80
Russian	13
German	12
Turkish	4
Farsi	3
Italian	2
Greek	2
Urdu	1
Norwegian	1
Japanese	1
*Total*	*491*

**Fig. 2:**
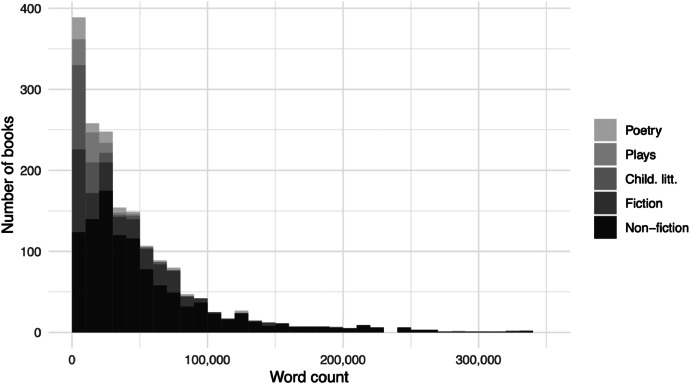
Word counts. Two books longer than 400, 000 words are excluded in the figure.

The length of works vary considerably, with children's literature, plays, and poetry being overrepresented in shorter books up to 30, 000 words. This is illustrated in Fig. 2.

One of the main benefits of this corpus is the representation of genres. Each book was labeled by category by the publisher from a list of 25 labels. Table 2 shows the representation of these genres in the corpus. Researchers may wish to combine these categories in various ways, depending on the research question.

**Table 2:** Counts and proportions of categories. Some books are assigned more than one category and are then listed more than once in the table. The sums of columns are therefore larger than the corpus totals.

Category	Words (millions)	% of tot.	Nr. of books
history	24.9	30.9	326
literature	18.6	23.1	340
novels	9.5	11.8	178
biographies	7.0	8.6	124
literary.criticism	6.5	8.1	93
science	6.5	8.0	175
philosophy	5.4	6.7	99
detective.fiction	4.8	5.9	169
travel.literature	3.6	4.4	55
social.sciences	3.8	4.7	65
poetry	3.5	4.4	89
psychology	1.0	1.3	19
politics	1.8	2.3	35
science.fiction	1.1	1.3	81
plays	1.3	1.6	88
children.stories	1.9	2.4	167
environmental.sciences	0.4	0.5	3
technology	0.5	0.6	3
geography	0.5	0.6	7
economics	0.4	0.5	10
arts	0.7	0.9	19
linguistics	0.8	0.9	22
health	<0.1	<0.1	1

### 3.2 File organization

The files in the repository are organized as follows:

corpus-txt/
  13028368.txt
  13035305.txt
  13050381.txt
  [...]
corpus-html/
  13028368.html
  13035305.html
  13050381.html
  [...]
metadata.tsv
README.md
README.md.pdf

Each book is identified with a unique eight-digit number assigned by the publisher. This number is used for file names for text files, as shown above. All files are in utf8 encoding.

The corpus is provided in two versions: html and unformatted plain text. These are found in the directories *corpus-html* and *corpus-txt* respectively. The plain-text version is suitable for most purposes.

The html-version may be of use for investigations requiring isolation of specific text elements. These files retain the markup from the xhtml files embedded in the original epub bundles. In addition to standard html markup for headers, paragraphs, italics, etc., the markup includes custom class elements (e.g., "<span class="quran"> ... </span>") . Some of these classes that may be of interest for researchers are:

• *quran*: Quotes from the Quran.
• *dialogue*: Dialogue in plays.
• *forignphrase* and *foreignphrase wordasword*: Non-Arabic script embedded in Arabic script.
• *poetry_container*: Poetry.

For example, searching for the quran tag in the corpus yields 10, 687 quotes from the Quran that can be extracted and analyzed in isolation.

The typeset, human readable versions of the books can be accessed from the URL http://www.hindawi.org/books/ followed by the eight-digit book number, or directly downloaded in various formats from the same URL and book number followed by ".pdf", ".epub", or ".kfx".

### 3.3 Metadata

Metadata for books in the corpus are provided in tab-separated format in the file metadata.tsv. This data file contains data for the following 24 variables:

(* = Only applicable to translated works. Empty string in non-translated works.)

• *booknr*: An eight-digit unique number assigned by the publisher, used in the corresponding file name.
• *category*: Category (genre) as assigned by the publisher. 213 books in the corpus have two or three labels, in which case these are given as a comma separated list. The category labels are listed in Table 2.
• *category.main*: Similar to category, but for books with two or three category labels, the second and third are omitted, leaving only the first and primary category label.
• *title*: The Arabic title of the book.
• origtitle*: The title of the original work.
• *author*: The author in Arabic script.
• origauthor*: The author in the original language.
• *pubdate*: Publication date of this edition (yyyy-mm-dd).
• *origpubdate*: Date of the original publication (yyyy).
• *origpubdate.full*: Similar to *origpubdate*, but for some books specified as
  - *yyyy-yyyy* (date range). Represented in *orgpubdate* by the first year in the rage.
  - *unkn*: Publication date stated in the work to be unknown. Represented in *orgpubdate* by "NA".
  - *mult*: Stated in the work to be originally published on multiple (unspecified) dates. Represented in *orgpubdate* by "NA".
• *translation*: Specifies whether the book is a translation from another language (TRUE/FALSE).
• transdate*: Translation date, provided for re-publications of existing translations (yyyy).
• *origlang.ar*: Arabic name of original language (e.g., "").
• *origlang*: English name of original language (e.g., "Arabic").
• *wc*: Word count.

## 4 Experimental Design, Materials and Methods

The data was collected and prepared as follows:

1) All 3, 485 books available as of April 4, 2024, were downloaded from the Hindawi website (http://www.hindawi.org) in epub format.
2) The ebup files were convert to html with Pandoc (http://www.pandoc.org). The resulting html files were then amended as follows:
  a. The <style>-tag specifying scss styling was removed.
  b. The files were indented for readability.
  c. The initial DOCTYPE declaration was replaced with the following (here broken into three lines for display) in order to be displayable in standard browsers:
    <!DOCTYPE html PUBLIC ``-//W3C//DTD XHTML 1.0
    Transitional//EN''
    “http://www.w3.org/TR/xhtml1/DTD/xhtml1-transitional.dtd”>
3) License information was extracted from the colophon in the html files and manually checked. Books published under non-free licenses were removed, including books where copyright is held by the author, the author's estate, or the publisher of the original publication. The remaining 1,745 books are either in the public domain or are published under CC BY 4.0 (https://creativecommons.org/licenses/by/4.0/).
4) Metadata (see above) were extracted from colophons in the corpus files and from the webpage for each book on the publisher's website. These data were extracted through an iterative process whereby a string was searched for to locate the relevant information. Books with no hit were identified and checked for a relevant string, which was then searched for in remaining relevant books, and so on, until all data for all books was located and extracted.
  For example, data on the original language of translated works were extracted by finding the string  (`This book was originally published in [the language]') in the colophons. This identified the original language for most of the translated works. For translated works where this string was not found, an alternative formulation giving this information was identified, which was then used to search in the remaining translated works, and so on, until the original language of all translated works was identified.
5) The html files were converted to plain text with Pandoc after removing (a) colophons (the contents of the <div class=“copyright”> tag) and (b) repetitions of the title from the cover, half title, and title page.

## Limitations

All texts in the corpus are from one single publisher.

## Ethics Statement

The current work does not involve human subjects, animal experiments, or any data collected from social media platforms. The paper complies with the ethical requirements for publication in *Data in Brief*.

## Credit Author Statement

Hallberg is the sole author of this paper.

## Data Availability

Swedish National Data SeriviceThe Arabic E-Book Corpus (Original data).

## Footnotes

1 The categories *Fiction* and *Non-fiction* in Figures 1 and 2 combine several categories specified in the metadata. See *Metadata* below.
